# Flight behavior of foraging and overwintering brown marmorated stink bug, *Halyomorpha halys* (Hemiptera: Pentatomidae)

**DOI:** 10.1017/S0007485315000462

**Published:** 2015-06-15

**Authors:** D.-H. Lee, T.C. Leskey

**Affiliations:** 1 Department of Life Sciences, Gachon University, 1342 Seongnamdaero, Sujeong-gu, Seongnam-si, Gyeonggi-do, Korea; 2 USDA-ARS, Appalachian Fruit Research Station, Kearneysville, West Virginia, USA

**Keywords:** invasive species, flight mill, dispersal, movement, pest management

## Abstract

Brown marmorated stink bug, *Halyomorpha halys* (Stål), is a highly polyphagous invasive species attacking both cultivated and wild plants increasing its threat to ecosystems as a global pest. However, dispersal biology of this invasive species is not well understood. This study evaluated the flight capacity and behavior of *H. halys* under laboratory, semi-field, and field conditions. Flight mills were used to measure the baseline flight capacity of adults collected year round from the field and included both foraging and overwintering populations. The effects of abiotic conditions such as wind speed and temperatures on the free flight parameters of *H. halys* were evaluated under semi-field and field conditions. The mean flight distances over a 22-h period were 2442 and 2083 m for male and female, respectively. Most individuals (89%) flew <5 km, though some flew much further with a maximum flight distance observed of 117 km. Flight distances by *H. halys* increased after emergence from overwintering sites in spring and reached their highest point in June. The incidence of take off by *H. halys* was significantly affected by the wind speed; when provided with still air conditions, 83% of individuals took off, but the rates decreased to <10% when wind speed was increased to or above 0.75 m s^−1^. The incidence of take off by *H. halys* was significantly affected by ambient temperature and light intensity in the field, whereas relative humidity and insect sex did not. When the temperature was at 10–15°C, 3% of individuals took off, but the proportion of *H. halys* taking flight increased to 61, 84, and 87% at 15–20, 20–25, and 25–30°C, respectively. In the field, prevailing flight direction was biased toward the opposite direction of the sun's position, especially in the morning. The implications of *H. halys* flight biology are discussed in the context of developing monitoring and management programs for this invasive species.

## Introduction

Brown marmorated stink bug, *Halyomorpha halys* (Stål), is a serious invasive species in the USA, especially in the mid-Atlantic regions. *H. halys* is native to China, Japan, Korea and Taiwan with over 100 host plants in 45 families (Hoebeke & Carter, 2003; Lee *et al.*, 2013*a*). This invasive species is highly polyphagous and has attacked both annual and perennial crops in the Eastern USA and poses an increasing threat in the Pacific Northwest of the USA, Canada and Europe (Leskey *et al.*, 2012; Gariepy *et al.*, 2014; Shearer & Wiman, 2014). This species is also problematic as a nuisance pest because a massive number of adults often invade human-made structures to overwinter inside (Inkley, 2012). *H. halys* also overwinter in natural landscapes using dry crevices in dead and standing trees, particularly *Quercus* spp. and *Robinia* spp. (Lee *et al.*, 2014).

As dispersal is critical to pest population dynamics (Stinner *et al.*, 1983; Jennersten, 1988; Kennedy & Storer, 2000), it is important to understand dispersal of target pest in order to develop and enhance monitoring and management tactics (Hughes & Dorn, 2002; Zhang *et al.*, 2009). Since initial discovery of *H. halys* populations in the mid-1990s in Allentown, PA, USA, it has quickly spread in the USA and now officially has been detected in 41 US states (http://www.stopbmsb.org for updates). This stink bug is an adept hitchhiker and often detected in vehicles and freight shipment (Holtz & Kamminga, 2010; Tindall *et al.*, 2012). Indeed, genetic evidence supports that North American populations of *H. halys* are likely derived from a single founding event (Gariepy *et al.*, 2014; Xu *et al.*, 2014). Human activity has played a role in the spread of this invasive species, but fundamental dispersal capacity of this insect also could be an important contributing factor.

Continuous tracking of *H. halys* movements in the field would be an ideal method for studying dispersal behavior and capacity. However, adult *H. halys* is a very active disperser with adults readily initiating rapid flights of over 100 m in <1 min when released in the field (Lee *et al.*, 2013*b*). Field observations also indicate that this insect often disperses among different habitats in pursuit of preferred hosts in the landscape, including its dispersal from overwintering sites to host plants in the spring (Lee *et al.*, 2013*a*). These factors make it difficult to measure *H. halys* flight parameters by tracking the same individual continuously. Therefore, experimentation with laboratory devices is an essential and practical step to understand dispersal of this highly mobile insect. Although there have been continuous efforts using flight mills in the laboratory to evaluate a variety of insects for their flight capacity (e.g., Krogh & Weis-Fogh, 1952; Hocking, 1953; Schumacher *et al.*, 2008), few studies (Jianxin & Wanzhi, 2009) have been conducted with Heteroptera. However, a recent study (Wiman *et al.*, 2014) demonstrated that flight mills were suitable devices to simulate free flight of *H. halys*. Wiman *et al.* (2014) reported clear dichotomy in the flight distance as 85% of test individuals flew 0–5 km (classified as ‘short distance fliers’ (SDFs)) over 24 h, whereas 15% flew >5 km (classified as ‘long-distance fliers’ (LDFs)) with the maximum flight distance of 75 km. *H. halys* were able to achieve flight speeds on the flight mills (Wiman *et al.*, 2014), comparable with speeds estimated from free flight in the field (Lee *et al.*, 2013*b*).

Flight mills are proven to be useful to evaluate baseline flight capacity of insects, but it can be challenging to extrapolate flight data gathered from tethered individuals to field behavior (Heinrich, 1971; Riley *et al.*, 1997; Taylor *et al.*, 2010). Thus, it is important to complement flight mill experiments with field trials whenever possible to acquire additional behavioral data. This effort would allow us to examine how much tethered insect flight may deviate from free flight behavior.

In this paper, we evaluated the flight behavior of *H. halys* in the laboratory and field. In particular, we collected *H. halys* year round from the field to measure dispersal capacity of actively foraging and settled overwintering populations in the laboratory flight tests. To our knowledge, this is the first attempt to measure flight capacity of overwintering insect populations by retrieving them directly from overwintering sites and testing them immediately. We also examined the flight behavior of field-collected *H. halys* under semi-field and field conditions to address how abiotic factors affect free flight parameters, including the likelihood of take-off and prevailing flight direction. We discuss the flight behavior and capacity of *H. halys* in the context of developing monitoring and management program for this insect.

## Materials and methods

### Flight mill bioassay

Wild *H. halys* were collected in the vicinity of Keedysville, MD from August 2012 to August 2013 at ~08:00 five times a week from the field. For foraging *H. halys*, adults were collected from cultivated and wild host plants at the University of Maryland Western Maryland Research and Education Center, Keedysville, MD from August to October 2012 and from June to August 2013. Host plants from which *H. halys* were collected included corn (58%), princess tree (20%), soybean (18%), and alfalfa (4%). For overwintering *H. halys*, adults were collected from a human-made overwintering site which was located <1 km from the collection sites used to obtain foraging *H. halys* individuals. Settled overwintering insects were collected from tight crevices between wooden boards and plastic tarps. Immediately after collection, adults were transported for ~30 min to the laboratory and then prepared for tethering to the flight mill at ~20°C for an additional 30–60 min prior to testing.

Six flight mills were assembled following the work by Smith (2012). Detailed information for flight mill construction (e.g., schematics, photographs, wiring diagrams, ordering information, and data acquisition programs) can also be found in Jones *et al.* (2010) and Wiman *et al.* (2014). The flight mills were housed in a laboratory room at 24°C, 41% RH, 1150 lux and a photoperiod of 14:10 (L:D). The DASYLab program (Measuring Computing, Norton, MA) was used to record the flight parameters such as the number of rotations per flight and flight duration. To tether *H. halys* on the flight mills, we treated the insects as follows. A droplet of glue from a low-temperature glue gun (3M Scotch-Weld Hot Melt Applicator LT, 3M, St. Paul, MN) was applied to the head of an insect pin and gently pressed onto the center of the *H. halys* pronotum. The point of the pin was inserted into the tip of one end of rotation arm of the flight mill, so that the insect could be positioned properly to allow flight. Trials were started ~09:30 by gently blowing each *H. halys* tethered on the mill to encourage flight. Thereafter, the insects were left in place undisturbed for 22 h. A total of 737 individuals were tested throughout the study period.

To allow for comparisons with *H. halys* populations from Oregon tested by Wiman *et al.* (2014), we used classifications designated by Wiman *et al.* (2014): individuals that flew 5 km or less were classified as ‘short distance fliers’ (SDFs) and those that flew more than 5 km as ‘long distanced fliers’ (LDFs). However, it is important to note that the two studies collected and classified test individuals in different ways. Wiman *et al.* (2014) collected *H. halys* adults exclusively on host plants from May to October and classified individuals into ‘overwintered’ vs. ‘summer’ populations. That is, adults that emerged from diapause in spring and began to forage were referred to as the ‘overwintered’ population, and their offspring and the potential subsequent generation adults are referred as the ‘summer’ population. In contrast, we collected *H. halys* adults year round and classified individuals into ‘overwintering’ vs. ‘foraging’ populations. Settled adults that were directly retrieved from overwintering sites are referred as the ‘overwintering’ population, and adults that were collected directly from host plants are referred as the ‘foraging’ populations.

Flight distances were compared among the months of trials (i.e., the months in which insects were collected) using a generalized linear model with Poisson distribution using log link function. The mean distances were further analyzed using Tukey's studentized range (HSD) test (PROC GENMOD, SAS 9.2, SAS Institute Inc., Cary, NC, USA). Preflight body weights of *H. halys* were compared between the group of interest (i.e., sex × population) using ANOVA with the Tukey's HSD test (PROC ANOVA, SAS 9.2, SAS Institute Inc., Cary, NC, USA). The proportions of body weight losses after flight were arcsine-transformed for the assumption of normality and analyzed using the ANOVA. The flight distances were log-transformed for normality and compared between the groups of interest including the preflight body weight as a covariate in the analysis (PROC GLM, SAS 9.2, SAS Institute Inc., Cary, NC, USA).

### Wind speed bioassay

Wild adult *H. halys* were collected from human-made overwintering sites from November to December 2012 in the vicinity of Kearneysville, WV. Insects were provided food, including potted soybean plants, peanuts, carrots, and sunflower seeds, and water in the laboratory at 25°C, 70% RH and a photoperiod of 16:8 (L:D) for >2 weeks to return them to an actively foraging stage.

A bioassay arena was set up in a greenhouse chamber (2.5 × 2.5 × 3 m^3^) at 25°C and 30% RH. A box fan (11 × 52 × 55 cm^3^; Lasko Products Inc., West Chester, PA) was placed on a table (~1 m tall) and a second fan was placed behind the first one facing the opposite direction. The first fan was used to generate airflow toward test insects and the second fan was used to reduce the wind speed projected from the first fan as needed. Using the two fans, it was possible to achieve six wind speed levels at ~0.7 m s^−1^ increments from 0.0 to 3.5 m s^−1^. Two bamboo dowels (1.2 m tall; 1 cm in diameter) were set up in parallel and 0.1 m apart; the two dowels were placed at 0.5 m away from the first fan. The probe of a hot-wire anemometer (AVM-714, Tecpel Co., Ltd., Taipei, Taiwan) was attached to the top of the first bamboo dowel. At the start of each experiment, a wind speed level was randomly chosen and the fans were calibrated appropriately. Wind speeds were recorded every 30 s during the trial. An adult *H. halys* was gently placed onto the second dowel ~30 cm from the dowel top. A previous study demonstrated that *H. halys* tend to climb the dowel and take flight in the field (Lee *et al.*, 2013*b*). After placing *H. halys* on the dowel, observations began and continued until the insect either took off or for a maximum of 3 min per trial. Trials were repeated with 40 individuals (sex ratio = 1:1) for each wind speed. Fisher's exact test was used to compare the proportion of *H. haly* taking flight among the wind speeds tested (PROC FREQ, SAS 9.2, SAS Institute Inc., Cary, NC, USA).

### Free flight bioassay

Wild *H. halys* were periodically collected from host plants such as soybean and *Paulownia tomentosa* trees in September 2012 in the vicinity of Kearneysville, WV. Insects were temporarily housed in field cages (1.8 × 1.8 × 1.8 m^3^) to enable test subjects to continue to experience natural environmental conditions. The cages were provisioned with food resources, including a potted peach trees, potted mullein plants, potted soybean plants, *Ailanthus* tree branches, *Paulownia* tree branches, and apples.

This experiment was conducted in an open plot covered by mowed grass (225 × 310 m^2^) at USDA-ARS, Appalachian Fruit Research Station, Kearneysville, WV. A bamboo dowel (1.3 m tall) was set up at the elevated point of the experimental plot (39° 21′ 27.6″N, 77° 53′ 26.0″W) with a minimum distance of 80 m to the plot borders. The dowel was used as release point of *H. halys* as described above. A previous study demonstrated that this approach provided a means to study take-off and flight behaviors of *H. halys*, including take-off rates, flight directions and speeds under field conditions (Lee *et al.*, 2013*b*). Temperature, humidity and light intensity were measured throughout the experiment to record abiotic conditions experienced by *H. halys*. At the onset of each trial, an adult *H. halys* (sex ratio = 1:1) was gently placed onto the bamboo dowel at ~30 cm from the dowel top. The trial began and continued until the insect either took flight or for a maximum of 3 min per trial. The data obtained for each individual included take-off incidence, time durations to take-off, and flight direction following a 30-m sustained flight. Observations were made between ~08:00 and 18:00 over 6 days (19–26 September, 2012) to include different abiotic conditions and times of day in the experiment. A total of 660 individuals were tested. The data were analyzed using the generalized linear model with binomial distribution to evaluate the significance of ambient temperature, relative humidity, light intensity, date, and sex as predictors in a single model on the incidence of take-off by *H. halys* (i.e., binomial response variables) (JMP 11, SAS Institute, SAS Institute Inc., Cary, NC, USA). Prevailing flight directions of *H. halys* were compared among five observation periods of day using Fisher's exact test (PROC FREQ, SAS 9.2, SAS Institute Inc., Cary, NC, USA).

## Results

### Flight mill bioassay

**Fig. 1.**
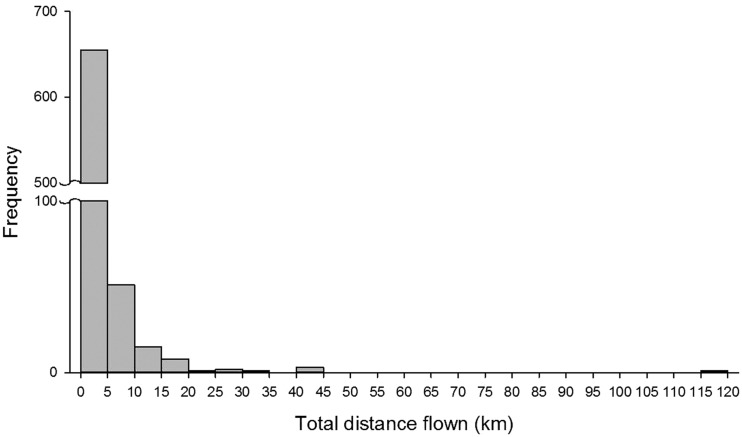
Frequency distribution of flight distance by *Halyomorpha halys* measured on flight mills for 22 h (*N* = 739).

Flight distance frequency for all *H. halys* individuals tested was highly aggregated at the distances of 0–5 km (fig. 1). This short distance flight group consisted of 88.9% of the all flights observed. Flight distances of 5–10 km represented 6.9%; all other flight distance groups contained <2.1% individuals tested. The mean flight distances were 2442 and 2083 m for male and female, respectively. For the foraging population, the maximum flight distances observed were 117 and 26 km for male and female, respectively. For the overwintering populations the maximum distances were 29 and 34 km for male and female, respectively.

**Fig. 2.**
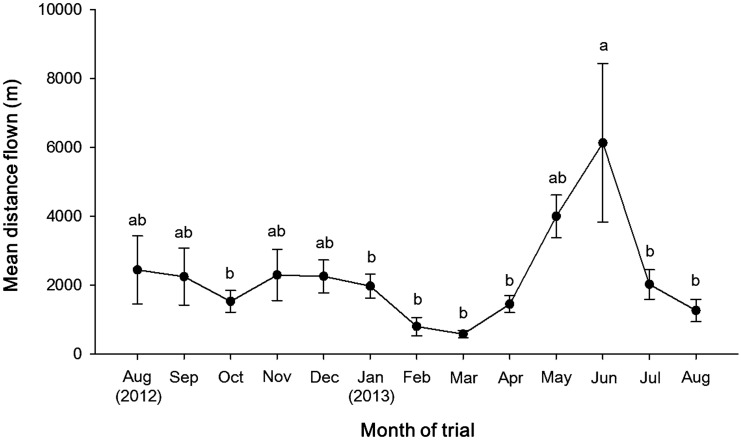
Mean flight distance by *Halyomorpha halys* (±SE) measured on flight mills from Aug 2012 through Aug 2013. Means followed by different letters were significantly different (*P* < 0.05).

The timing of *H. halys* collections significantly affected the mean flight distance by the insects along the Gregorian calendar (χ^2^ = 611, 648, *P* < 0.0001) (fig. 2). The flight distances remained at relatively constant levels between August and October 2012 from which foraging F_1_ and F_2_ generation *H. halys* were collected from host plants. Overwintering *H. halys* yielded similar levels of flight distances through January, but flight distance by overwintering populations reached the lowest points in February through March. Flight distances sharply increased in May through June as foraging adults were collected in the field. Then, the flight distances decreased to the similar levels of those measured in August of the previous year.

**Fig. 3.**
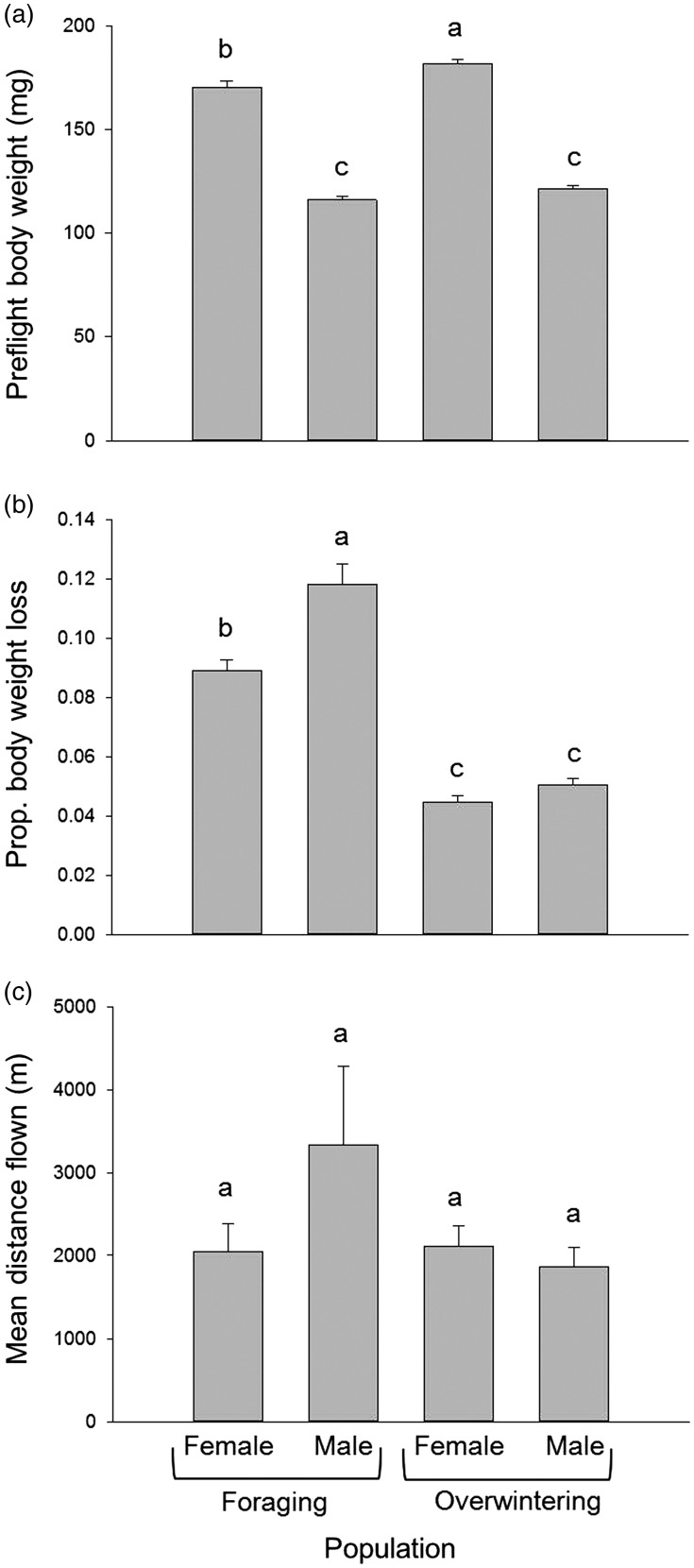
(a) Mean preflight body weight of *Halyomorpah halys* (±SE); (b) proportion of body weight loss of *H. halys* (±SE) after the 22-h flight mill test compared to the preflight weight; and (c) mean flight distance by *H. halys* (±SE) measured on flight mills. Means followed by different letters were significantly different (*P* < 0.05).

Overall, females were significantly heavier than males, and females from overwintering populations showed the heaviest body weight before flight (*F*_3,733_ = 305.00, *P* < 0.0001) (fig. 3a). The proportion of body weight losses of foraging populations was significantly greater than overwintering populations for either female or male (*F*_3,642_ = 106.72, *P* < 0.0001) (fig. 3b). The body weight losses were significantly greater for male than female within foraging populations (*P* < 0.05); however, this pattern was not the case for overwintering populations. Although the mean flight distances by foraging males were numerically greater than others, there was no detectable significant difference among four test groups due to high variability of the data within test group (e.g., foraging male) (*F*_7,729_ = 1.86, *P* = 0.0739) (fig. 3c).

### Wind speed bioassay

**Fig. 4.**
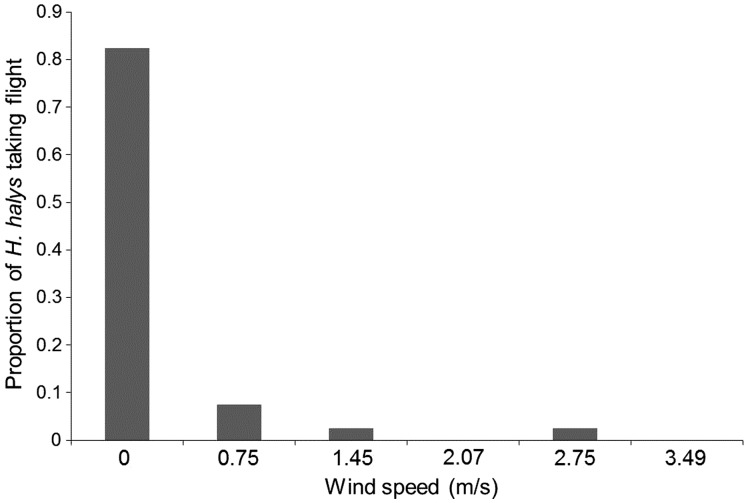
Proportion of *Halymorpha halys* taking flight across the wind speeds generated in a greenhouse experimental arena.

The proportion of *H. halys* taking flight was significantly affected by the wind speed generated in the semi-field experimental arena (Fisher's exact test, *P* < 0.0001) (fig. 4). The probability of taking flight exponentially decreased as the wind speed increased. When given still air conditions, 83% of *H. halys* took off within 3 min. However, the probability of taking flight decreased to <10% at or above the wind speed of 0.75 m s^−1^ (fig. 4).

### Free flight bioassay

**Fig. 5.**
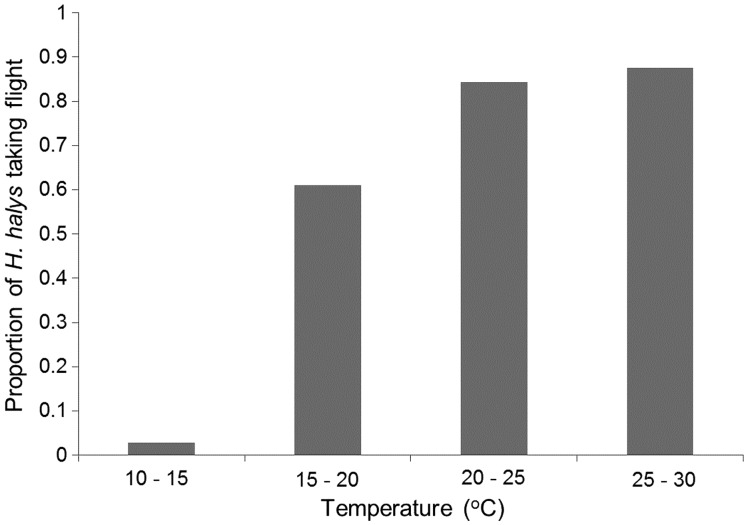
Proportion of *Halymorpha halys* taking flight across the ambient temperatures measured in the field conditions.

**Fig. 6.**
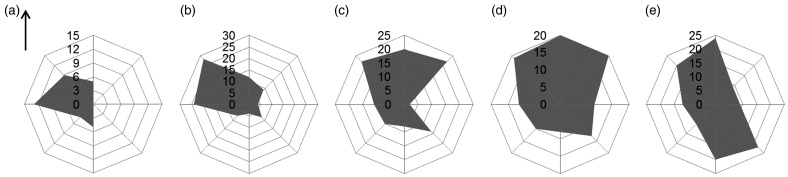
Prevailing flight directions of *Halymorpha halys* observed in the field conditions at (a) 8:00–10:00, (b) 10:00–12:00, (c) 12:00–14:00, (d) 14:00–16:00, and (e) 16:00–18:00. Arrow indicates the north.

The proportion of *H. halys* taking flight was significantly affected by ambient temperature and light intensity in the field (*P* < 0.05) (table 1). No effects were observed from relative humidity, date of observation or sex (table 1). Temperature was the most significant factor (*P* < 0.0001) (table 1), and thus the proportion of *H. halys* taking flight is presented in fig. 5 across the temperature range observed in the field. When the temperature was at 10–15°C in the field, 3% of *H. halys* took off from the bamboo dowel. However, the proportion of *H. halys* taking flight increased 20 times, reaching at 61%, at the temperature range of 15–20°C. At higher temperature ranges, the proportions of *H. halys* taking flight were 84 and 87% at 20–25 and 25–30°C, respectively. The prevailing direction of *H. halys* flight was significantly affected by the time of day (Fisher's exact test, *P* < 0.0001) (fig. 6). In general, the prevailing flight direction was biased toward the opposite directions of the sun's position in the morning; though this pattern became less conspicuous in the afternoon.

**Table 1.** The statistical significance of abiotic conditions and insect sex on the incidence of take off by *Halyomorpha halys* tested in a single generalized linear model.

Factor	Chi-square	d.f.	*P*
Ambient temperature	26.386	1	<0.001
Relative humidity	3.207	1	0.073
Light intensity	6.447	1	0.011
Date	8.666	5	0.123
Sex	1.831	1	0.176

## Discussion

This study utilized both laboratory and field approaches to better understand flight behavior and capacity of *H. halys*. The results of this study indicate that *H. halys* has strong flight capacity with which populations are capable of expanding their invasion into new regions at landscape levels. This study also provides information about abiotic conditions by which flight behaviors of *H. halys* can be significantly affected in the field. This knowledge will be useful toward development of monitoring and management tools for this invasive species.

The flight mill system has proven very useful to measure baseline flight capacity of *H. halys* as previously reported by Wiman *et al.* (2014). The results of this study are consistent with the previous study (Wiman *et al.*, 2014) in many aspects, although there are also differences in the results between the two studies. As mentioned above, our study confirms that both foraging and overwintering populations have substantial flight capacity. Foraging populations flew 2.7 km on average with 13% LDFs flying >5 km in the populations. Similar levels of flight distance and the proportion of LDFs was observed from foraging *H. halys* populations collected in Oregon (Wiman *et al.*, 2014). In addition, flight velocity of *H. halys* measured in the two studies was similar and comparable with the flight speed measured from free flight in the field (Lee *et al.*, 2013*b*).

However, it is noteworthy that the effect of timing of *H. halys* collection on flight distance was quite different between the two studies. Wiman *et al.* (2014) collected *H. halys* adults exclusively from host plants from early May through early October in Oregon and found that the flight capacity was initially high before reaching the lowest point during mid-season (i.e., June–July). By contrast, this study found that foraging *H. halys* had increased the flight capacity after emergence from overwintering sites and reached their highest point in June. The difference between the two studies may result from several reasons. One difference comes from experimental designs used in the two studies. Wiman *et al.* (2014) started collecting *H. halys* adults from host plants at the beginning of May, but our study collected *H. halys* adults from overwintering sites until the end of May. In May, very few *H. halys* were found foraging on host plants in/around the collection sites in Maryland. Also, Wiman *et al.* (2014) collected *H. halys* mainly from ornamental plants, including English holly trees (76%), native and ornamental maples (6%), and lilac bushes (3%), whereas we collected the insects mainly from corn (58%), *Paulownia* (20%), and soybean (18%). Nutritional and phenological traits of host plants that *H. halys* had been foraging could have affected the flight capacity and the pattern of its change in the studies. In addition to the experimental designs mentioned above, we cannot rule out potential effects of climates on the phenology and biological traits of *H. halys* populations in the two geographically separated locations. A recent study monitored the emergence patterns of overwintering *H. halys* in the field and found that the peak dispersal occurs in late May to early June in the mid-Atlantic regions (J. C. Bergh & T. C. Leskey, unpublished data).

This study measured the flight capacity of overwintering populations using the flight mill systems with the same methodology used for the foraging populations. To our knowledge, this is the first study reporting flight capacity of overwintering pentatomids by retrieving test individuals directly from overwintering sites. The retrieved overwintering individuals were immediately brought to the laboratory after collection and tethered on the flight mills for the test. It is striking that overwintering *H. halys* readily flew 2 km per 22 h and 10% individuals were LDFs flying >5 km. Especially, given that the flight capacity of overwintering *H. halys* sharply increased in May during which *H. halys* tends to start emerging from overwintering sites; this represents a high risk of long-distance dispersal of *H. halys* to available host plants. Potential management programs for *H. halys* could target this overwintered population to prevent them from dispersing and establish them during the early season. In addition, it has been demonstrated in the field that overwintered *H. halys* adults were more vulnerable to insecticide toxicity than F_1_ and F_2_ generations (Leskey *et al.*, 2014).

Males outperformed females of foraging populations in overall flight distance in our studies. The result is rather consistent with general patterns observed from other insects such as the red milkweed beetle; males in general are more vagile than females, thereby maximizing their likelihood of mating with as many females as possible (Davis, 1984). This difference was not observed from our overwintering populations supporting the reasoning for the observed pattern. Interestingly Wiman *et al.* (2014) reported that females demonstrated greater flight capacity compared with males. Wiman *et al.* (2014) hypothesized that the aggregation pheromone of *H. hays*, which is produced only by sexually mature males, might have resulted in sex-role reversal and that females may need to increase their flight capacity to maximize mating opportunity by finding pheromone-producing male groups and host plants simultaneously for oviposition (Wiman *et al.*, 2014).

The proportion of body weight loss was greater for males than females of the foraging population; however, there was no significant difference between males and females from the overwintering populations. Given that males flew greater distances than females of the foraging populations, the body weight losses might have mainly resulted from the energy use for flight. Overall, the proportion of body weight loss of the overwintering population was >50% lower than that of the foraging population. As *H. halys* accumulate a large amount of fat body before entering into overwinter stage (A. L. Nielsen, unpublished data) and the fat body is the main resource for overwintering survival (Funayama, 2012), it is likely that overwintering *H. halys* had experienced less body weight loss compared with foraging individuals which mainly used carbohydrate-based energy resources for flight. Further studies are warranted to examine nutritional effects on the physiology and energy expenditure of *H. halys*.

This study reveals that abiotic conditions such as wind speed and temperature significantly affect the likelihood of *H. halys* to take flight. This information is essential to establishing a better understand flight biology of *H. halys* along with the baseline flight capacity measured using the flight mill system. The semi-field experiment indicates that even very mild wind such as at the wind speed of 0.75 m s^−1^ substantially decreases the proportion of *H. halys* to take off compared with still air. Thus, it would be useful to monitor *H. halys* on crops especially during warm and calm days, which may encourage more *H. halys* movement between plots and crops. This may also affect *H. halys* captures by monitoring traps such as pheromone or light traps. The speed of 0.75 m s^−1^ is classified as ‘light air’ or ‘force 1’ out of 13 levels at the Beaufort wind scale; smoke starts drifting according to wind direction at this wind speed level. Therefore, it is expected that *H. halys* would take flight over limited conditions during which microclimates provide virtually still air around the insect.

In the field, ambient temperature was the most significant factor affecting the likelihood of *H. halys* to take flight over a day. The direct observations on *H. halys* flight were conducted between 08:00 and 18:00 in September, when the temperature typically increased from 10 to 30°C over the observation period. When temperatures were below 15°C, only 3% of *H. halys* made flight, but the insects increased the incidence of taking flight at least by 20 times when temperatures were above 15°C. This information can enhance management programs for *H. halys* such as insecticide applications. If insecticide applications were made under the critical temperature for *H. halys* flight, then the insects would be less likely to disperse from treated areas. In addition, the results of this study indicate that prevailing directions of *H. halys* flight changes over a day generally toward the opposite direction from the sun's position, especially in the morning. This information can be useful in predicting the local movement patterns of *H. halys* between crop fields and surrounding wild hosts by considering the diurnal directionality of *H. halys* flight.

Some studies have also reported variations in flight performance of other Hemipteran species in relation to their biological traits and abiotic factors. Dingle *et al.* (1980) evaluated flight performance of milkweed bugs, the genus of *Oncopeltus* (Hemiptera: Lygaeidae) using a tethered flight technique. The study found significant differences among species and populations, where in general there was an association between larger body size and longer flight (Dingle *et al.*, 1980). Another study revealed that it is critical to test field-collected populations, as opposed to laboratory populations, to accurately estimate the flight performance (Baker *et al.*, 1980). Field-collected populations of *Nilaparvata lugens* (Stål) (Hemiptera: Delphacidae) flew longer and more readily compared with laboratory-bred insects (Baker *et al.*, 1980). Indeed, laboratory-reared *N. lugens* performed poorly even after only one generation in the laboratory environments. Lu *et al.* (2007) evaluated the effects of age, sex and mating status of *Lygus lucorum* (Meyer-Dür) (Heteroptera: Miridae) on flight potential using a flight-mill system. In the study, 10-day-old mated females yielded the greatest flight distance (40.1 km) and duration (7.7 h) in 24-h flight assays).

This study confirms that *H. halys* has baseline capacity to make long-distance dispersal (e.g., >5 km) over a day. Results indicates ca. 10% of individuals in the population have this capacity. Pentatomidae are known to disperse in the pursuit of host plants as they mature and senesce at landscape levels (McPherson & McPherson, 2000). Therefore, phoresy is not the sole factor responsible for the spread of this insect at various landscape levels. Observational studies on free flight also provide useful information to quantify the probability *of H. halys* to make flight along abiotic conditions. This knowledge should serve as foundation to develop monitoring and management programs for this invasive species.
